# Wind and structural loads data measured on parabolic trough solar collectors at an operational power plant

**DOI:** 10.1038/s41597-023-02896-4

**Published:** 2024-01-19

**Authors:** Ulrike Egerer, Scott Dana, David Jager, Geng Xia, Brooke J. Stanislawski, Shashank Yellapantula

**Affiliations:** National Renewable Energy Laboratory (NREL), Golden, CO 80401 USA

**Keywords:** Solar thermal energy, Atmospheric dynamics, Mechanical engineering, Fluid dynamics

## Abstract

Wind loading is a primary contributor to structural design costs of concentrating solar-thermal power collectors, such as heliostats and parabolic troughs. These structures must resist the mechanical forces generated by turbulent wind, while the reflector surfaces must maintain optimal optical performance. Studying wind-driven loads at a full-scale, operational concentrating solar-thermal power plant provides insights into the wind impact on the solar collector field beyond the capabilities of wind tunnel tests or state-of-the-art simulations. We conducted comprehensive field measurements of the atmospheric turbulent wind conditions and the resulting structural wind loads on parabolic troughs at the Nevada Solar One plant over a two-year period. The measurement setup included meteorological masts and structural load sensors on four trough rows. Additionally, a lidar scanned the horizontal plane above the trough field. In this study, we describe the high-resolution dataset characterizing the complex flow field and resulting structural loads. This first-of-its-kind dataset will enhance the understanding of wind loading on collector structures and will help in designing the next-generation solar collectors and photovoltaic trackers.

## Background & Summary

Concentrating solar-thermal power (CSP) presents a promising approach to harness solar energy for both electricity generation and industrial heat applications, with the added advantage of providing thermal energy storage. CSP systems employ specialized solar collectors like heliostats and parabolic troughs, which concentrate sunlight onto tower-based receivers or linear receiver tubes, respectively. The concentrated solar heat is then used for various energy generation or heat-intensive processes.

The solar collectors constitute almost one-third of the total cost of the power plant. One of the primary drivers of reliability issues in these collectors is the wind-driven loading of mirrors, support structures, and drives. The significance of wind loads in collector design is emphasized in NREL’s *Concentrating Solar Power Best Practices Study*^1^ as well as the heliostat roadmap^2^. Complex dynamic wind conditions in a collector field and the corresponding loads on structural components are not well understood, but they affect structural lifetime, optical performance, and costs of collectors^3^. For example, wind speeds may decrease in the interior of the trough field while turbulence intensifies^4^ with an uncertain effect on dynamic structural loads. Currently, the design of solar collector structures has predominantly relied on data from wind tunnels^5,6^. Early wind tunnel studies^7–9^ gathered fundamental knowledge about structural loads on the collectors caused by different wind conditions. However, wind tunnel experiments can only model a limited part of the turbulent energy spectrum^10^ and do not adequately capture the dynamic effects observed at scale. Full-scale measurements have been performed on single solar collectors^11,12^ or smaller test fields^13^ and have shown wind modifications over several troughs in limited configurations. Further, the wind field in a collector field has been reconstructed by means of numerical flow simulations^14,15^, and structural responses to wind have been simulated with finite-element analysis^14,16^ in idealized conditions. However, combined measurements of wind conditions and structural loads in a full-scale collector field are not available to date. Additionally, there exists a critical gap in knowledge regarding the deformation and deflections of structural members in mirror assemblies under wind loads. These deformations and deflections can significantly impact the optical performance of solar collectors, thereby reducing the overall plant efficiency.

To generate a comprehensive dataset of wind loading on collector structures, we performed an extensive measurement campaign at an operational parabolic trough power plant. The aim was a detailed characterization of prevailing wind and turbulence conditions and resulting operational loads on parabolic troughs to provide insights into structural dynamic response. NREL initiated the field measurement campaign at the Nevada Solar One (NSO) parabolic trough plant (https://solarpaces.nrel.gov/project/nevada-solar-one). Operating since 2007, the plant has a nominal capacity of 72 MW with 0.5 h storage, and uses thermal oil as the receiver working fluid at temperatures up to 393 °C. Constituting the solar collector field, parabolic troughs are aligned in the north-south direction and track the sun from east to west over the course of a day, facing varying wind loads depending on the wind properties and the angle of the troughs. The collectors (type *SGNX-1*) are designed as an aluminum space frame construction with glass mirrors. During strong winds and nighttime, the troughs are stowed 30° below the horizon to the east.

The measurements at NSO, running from October 2021 through June 2023, were recorded by sonic anemometers on masts at different heights to characterize the incoming flow and conditions around four trough rows at the western edge of the trough field. In addition, a Doppler lidar scanned the horizontal plane above the troughs. These wind measurements were combined with structural load measurements. The load measurements were installed on the same four outermost trough rows and included support structure bending moments, drive torque moments, dynamic accelerations of the space frame, mirror displacement, and tilt angles. Our measurements stand out for several reasons. First, they encompass a combination of wind conditions across multiple trough rows, along with corresponding loads and the wind field directly above the trough field. Second, our measurements extend over a long time period, allowing us to capture a broad variation in environmental conditions. Last, we employed high-frequency measurements, enabling us to study the dynamic interactions and intricate dynamics of the system in detail. The present paper describes the extensive field measurements of the wind masts, structural loads, and lidar. In addition to using these data for designing next-generation solar collectors, the dataset can be used to create and validate computational models for predicting the unsteady flow conditions and wind loading in collector arrays.

## Methods

### Overview of the measurements at Nevada Solar One

**Fig. 1:**
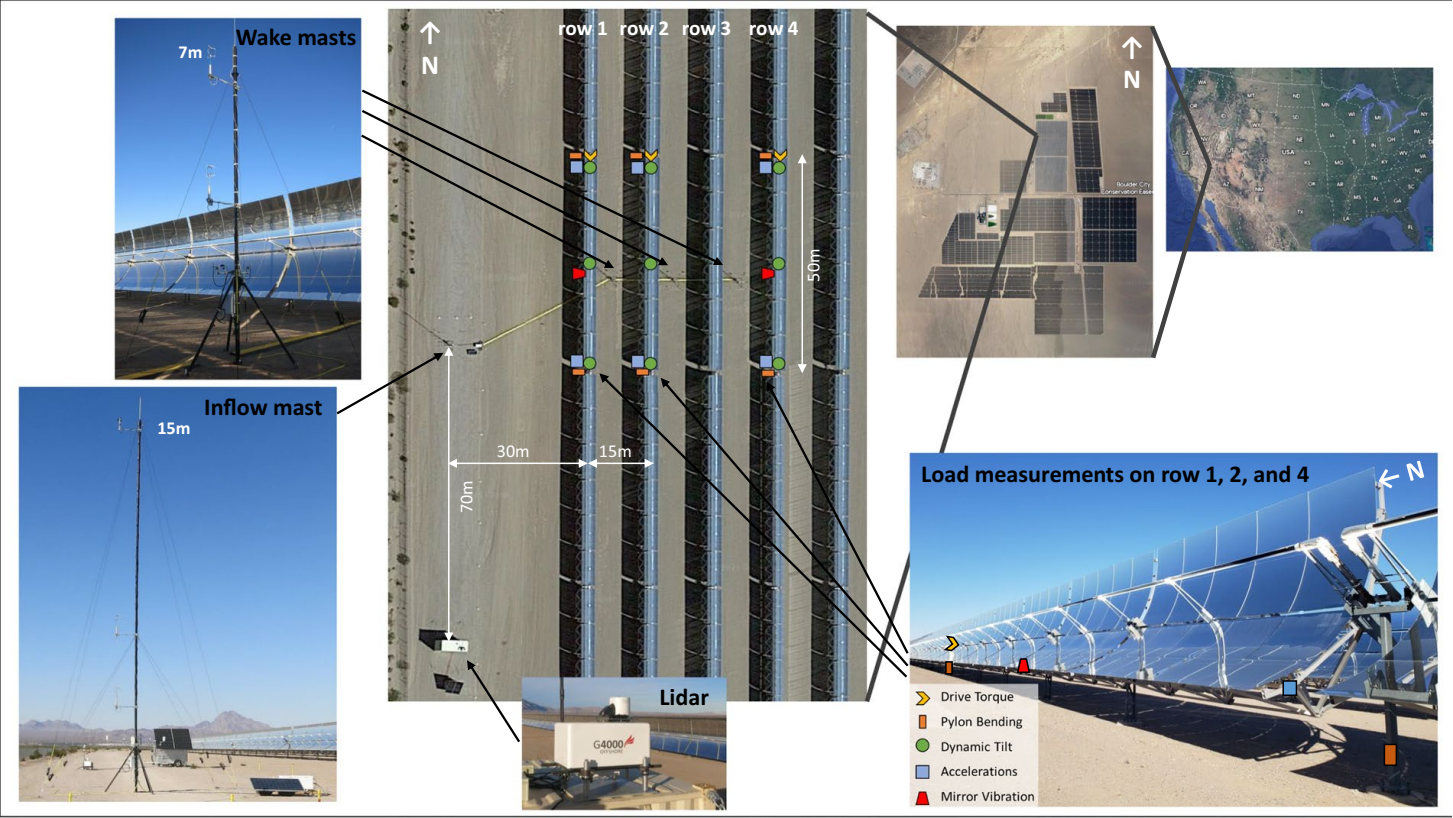
Overview of wind and structural loads measurements at NSO. *Satellite images: © 2023 Google Earth Data*.

**Fig. 2:**
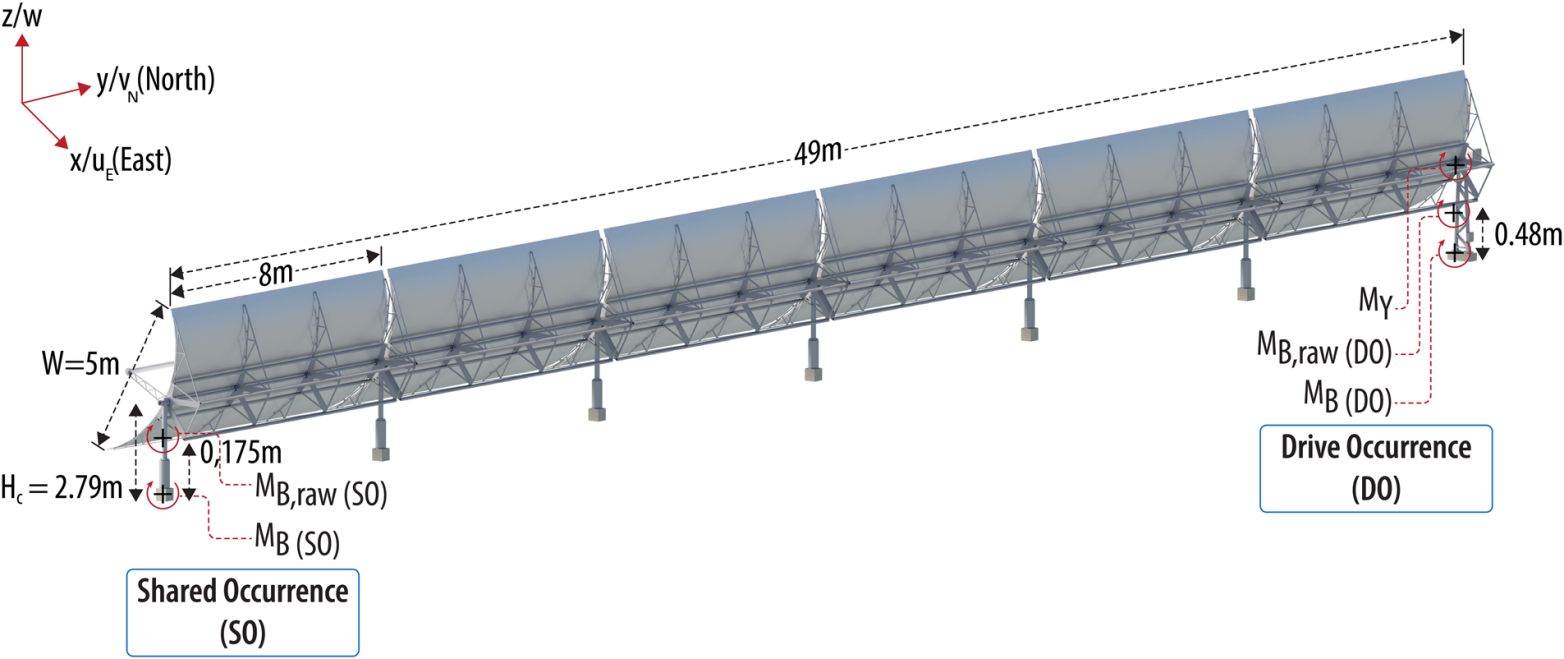
Bending and torque moment definitions at the drive occurrence (DO) and shared occurrence (SO) with trough dimensions and the loads/wind coordinate system. The sketch shows one half of an SCA. *Image courtesy Besiki Kazaishvili, NREL*.

**Fig. 3:**
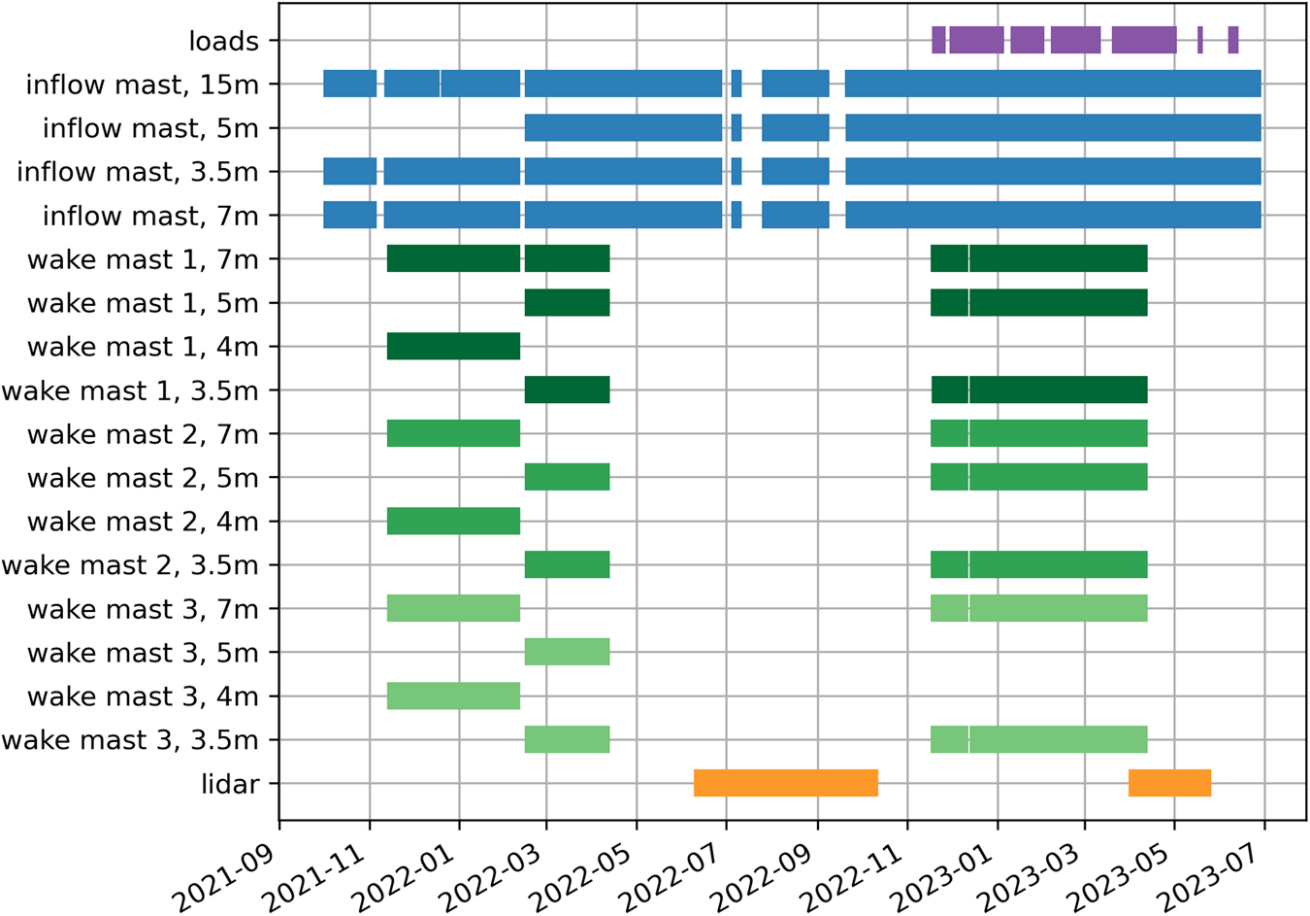
Data availability throughout the measurement period at NSO.

The NSO parabolic trough plant is located near Boulder City, Nevada, USA, at 35.8^◦^N, −114.983^◦^E and at 540 m elevation in a hilly desert environment. The plant’s solar collector field consists of 95 loops, each with eight 100 m long solar collector assemblies (SCAs) aligned in the north-south direction. NREL’s measurement setup is located at the western edge of the collector field at the four outermost rows, targeting winds perpendicular to the trough rows. Figure 1 shows an overview of the instrumentation setup. Meteorological masts for in situ wind measurements and load sensors on the collector structures are strategically installed within 49 m long half-sections of an SCA (Fig. 2). The trough angle of each collector assembly is controlled by a drive actuator in the center of the assembly (the northern end of our measured segment), situated on the drive occurrence (DO) pylon. The loose bearing at the southern end is referred to as the shared occurrence (SO) pylon. More details about the trough geometry are given in the section about structural loads measurements. The lidar is located 70 m south of the inflow mast outside of the field. Figure 3 summarizes the time periods of data availability for the different instruments. The masts continuously operated between October 2021 and June 2023. Due to regular maintenance on the parabolic troughs between April 2022 and November 2022, the NREL team decommissioned the wake towers between the rows in this time period. Structural loads measurements were added in November 2022. The lidar operated throughout the entire period in different scanning configurations, but only the periods of published data are shown in Fig. 3.

### Mast measurements of winds

#### General setup

The mast setup consists of an inflow mast outside of the array and three wake masts between the four westernmost trough rows. Table 1 and Fig. 3 provide an overview of the individual instrument characteristics on all masts and their operating period. The inflow mast with sonic anemometers at heights of 7 m, 5 m, and 3.5 m characterizes the incoming flow on the western side of the trough field. The sonic anemometers provide wind speed components in the north, east, and upward directions as well as sonic temperature with a temporal resolution of 20 Hz. The 7 m sonic anemometer measurements extend above the parabolic troughs, and the 3.5 m sonic anemometer is located approximately at the hinge height of the troughs. A cup anemometer adds the horizontal wind speed at 15 m height. At 2 m height, temperature, relative humidity and barometric pressure were measured at 1 Hz frequency. The configuration was slightly changed over the course of the measurement period: The 5 m sonic anemometer was added first in February 2022, the lowest sonic anemometer had to be replaced twice due to availability of the instruments, and the temperature sensors at 3.5 m and 7 m were first added in November 2022.

**Table 1:** Instrumentation for the inflow and wake masts.

Height	Measured quantity	Instrument	Model
**Inflow mast**			
15 m	*U* (1 Hz)	Cup anemometer	Thies First Class Advanced
7 m	*u*_*E*_, *v*_*N*_, *w*, *T*_*s*_	Sonic anemometer	Gill WindMaster Pro
	*T* (1 Hz)	PT100	Rosemount 68 RTD (after Nov 2022)
5 m	*u*_*E*_, *v*_*N*_, *w*, *T*_*s*_	Sonic anemometer	Gill WindMaster
3.5 m	*u*_*E*_, *v*_*N*_, *w*, *T*_*s*_	Sonic anemometer	Gill WindMaster (to Feb 2022)
			Gill WindMaster HS (Feb 2022 to Nov 2022)
			Campbell CSAT-3 (after Nov 2022)
	*T* (1 Hz)	PT100	Rosemount 68 RTD (after Nov 2022)
2 m	*T*, *p*, RH (1 Hz)	Capacitive sensor	Vaisala PTU307
	GPS time	GPS receiver	Garmin GPS16X-HVS
**Wake mast 1**			
7 m	*u*_*E*_, *v*_*N*_, *w*, *T*_*s*_	Sonic anemometer	Gill WindMaster
5 m	*u*_*E*_, *v*_*N*_, *w*, *T*_*s*_	Sonic anemometer	Gill WindMaster
3.5 m/ 4 m	*u*_*E*_, *v*_*N*_, *w*, *T*_*s*_	Sonic anemometer	Gill WindMaster (to Feb 2022)
			Gill WindMaster HS (Feb 2022 to April 2022)
			Campbell CSAT-3 (after Nov 2022)
**Wake mast 2**			
7 m	*u*_*E*_, *v*_*N*_, *w*, *T*_*s*_	Sonic anemometer	Gill WindMaster
5 m	*u*_*E*_, *v*_*N*_, *w*, *T*_*s*_	Sonic anemometer	Gill WindMaster
3.5 m/ 4 m	*u*_*E*_, *v*_*N*_, *w*, *T*_*s*_	Sonic anemometer	Gill WindMaster (to Nov 2022)
			Campbell CSAT-3 (after Nov 2022)
**Wake mast 3**			
7 m	*u*_*E*_, *v*_*N*_, *w*, *T*_*s*_	Sonic anemometer	Gill WindMaster
5 m	*u*_*E*_, *v*_*N*_, *w*, *T*_*s*_	Sonic anemometer	Gill WindMaster
3.5 m/ 4 m	*u*_*E*_, *v*_*N*_, *w*, *T*_*s*_	Sonic anemometer	Gill WindMaster

Additionally, three wake masts between the four westernmost rows characterize the wind field between the trough rows with sonic anemometers at heights of 3.5 m, 5 m, and 7 m. Again, the configuration was slightly modified due to practical reasons–most importantly, the height of the lowest sonic anemometers was changed from 4 m to 3.5 m in February 2022. The 5 m sonic anemometer on mast 3 was not installed in the period starting November 2022.

Prior to installation, all meteorological instruments were calibrated. The cup anemometer and Gill WindMaster sonic anemometers were calibrated in an accredited wind tunnel, while the CSAT3 sonic anemometers and Gill WindMaster HS sonic anemometers (new units) were calibrated at the manufacturer. The NREL calibration lab, an ISO 17025-accredited facility, calibrated the Vaisala PTU (pressure, temperature, and humidity) sensors in an environmental chamber and the Rosemount temperature sensors in an oil bath.

A Campbell CR1000X data logger collected the digital input signals of all met mast instruments and a GPS receiver. The GPS time stamp was used for synchronization with other data streams. Data were transferred to NREL servers hourly using the data logger server software via a cellular modem. The program’s primary scan, at 20 Hz, was used to poll the sonic anemometers with secondary scans at 1 Hz for the GPS synchronization, cup anemometer, temperature, humidity and pressure sensor. The GPS ensured the real-time clock accuracy was within ± 10 *μ* s. Raw data were sent to an NREL server and stored as hourly files separately for the inflow 20 Hz and 1 Hz data and for each wake mast. These files were then used for postprocessing.

#### Data processing

In the data postprocessing routines, the hourly raw data files are read and merged into daily datasets for the inflow mast and combined wake masts. The general processing steps for all sonic anemometers are identical. For all parameters, outliers are excluded by detecting values outside a 5-times standard deviation (5σ) in a 60 s rolling window. The amount of detected outliers is consistently below 2% of daily data for all sonic anemometers. The wind coordinate system in the raw sonic data, which is based on the specific sonic model, is transformed into a right-handed local tangent plane coordinate system with wind speed components *u*_*E*_ in the east direction perpendicular to the parabolic trough rows, *v*_*N*_ in the north direction along the trough rows, and upward *w* (“ENU”). This system is also consistent with the loads coordinate system. All Gill sonic anemometers we affected by the “w-boost” firmware bug^17^, which includes an underestimation of measured vertical wind speeds. We apply the correction factors of 1.166 for upward velocities and 1.289 for downward velocities to the raw data. Then, the horizontal wind speed *U* is calculated by

1 $$U=\sqrt{{u}_{E}^{2}+{v}_{N}^{2}}$$

and the meteorological wind direction WD (indicates where wind is coming from) results from

2 $${\rm{WD}}=\arctan \left(\frac{{u}_{E}}{{v}_{N}}\right)+\frac{\pi }{2}$$

with respect to the quadrant of *u*_*E*_ and *v*_*N*_ in the defined coordinate system. The sonics in the field were oriented with a calibrated digital protractor; no further tilt corrections were made due to the complexity of the surrounding environment. The low-resolution meteorological data, including temperature *T*, pressure *p*, and relative humidity (RH) at 2 m height, are added to the sonic wind speed data. The sonic output includes high-frequency measurements of sonic temperature (which is approximately equal to virtual temperature); however, these sonic temperature values are not calibrated. No consistent calibration has been found for the time period when actual temperature data at the sonic height levels were available. Despite this limitation, the sonic temperature measurements still capture high-frequency variations in temperature, which are used for covariance estimations (see below). The separate temperature sensors installed at the inflow mast yield more accurate absolute temperature values, although with a larger response time. Additionally, for each sonic anemometer, the turbulence parameters turbulence kinetic energy (TKE) and turbulence intensity (TI) are calculated in a running 10 min time window for each time step, ensuring a high resolution of TI and TKE. The TKE is determined by summing the squares of the velocity standard deviations in the three orthogonal directions:

3 $${\rm{TKE}}=0.5\cdot ({\sigma }_{uE}^{2}+{\sigma }_{vN}^{2}+{\sigma }_{w}^{2})$$

where *σ*_*uE*_, *σ*_*vN*_, and *σ*_*w*_ represent the standard deviations of the wind velocity components. The TI along the mean horizontal wind $\overline{U}$, for the vertical wind component *w*, and for the wind components perpendicular to the troughs *u*_*E*_ and along the troughs *v*_*N*_, is calculated as the ratio of the standard deviation of the respective wind speed component to the mean wind speed:

4 $${{\rm{T}}{\rm{I}}}_{U}=\frac{{\sigma }_{U}}{\bar{U}}\,,\,{{\rm{T}}{\rm{I}}}_{w}=\frac{{\sigma }_{w}}{\bar{U}}\,,\,{{\rm{T}}{\rm{I}}}_{uE}=\frac{{\sigma }_{uE}}{\bar{U}}\,\,{\rm{a}}{\rm{n}}{\rm{d}}\,\,{{\rm{T}}{\rm{I}}}_{vN}=\frac{{\sigma }_{vN}}{\bar{U}}\cdot $$

These turbulence parameters provide insights into the dynamic characteristics and intensity of turbulence within the measured wind field and in relation to the array of parabolic troughs.

At the inflow mast, additional parameters are derived to characterize the state of the atmospheric boundary layer. To assess atmospheric stability, the bulk Richardson number is calculated between the 3.5 m and 7 m height level, for time periods with temperature measurements at these levels. The bulk Richardson Number (Ri_*b*_) is defined by:

5 $$R{i}_{b}=\frac{g}{{\overline{T}}_{{\rm{ref}}}}\cdot \frac{\Delta T\cdot \Delta z}{{(\Delta {u}_{E})}^{2}+{(\Delta {v}_{N})}^{2}}$$

where *g* = 9.81 m s^−2^ is the acceleration due to gravity, *T*_ref_ is the average temperature between the two levels, and Δ*z* = 3.5 m is the height difference with differences in temperature Δ*T* and in the horizontal wind speed components Δ*u*_*E*_ and Δ*v*_*N*_. As part of the surface energy budget, turbulent energy fluxes describe the energy transport by turbulent eddies within the atmospheric boundary layer. These fluxes are directly determined from our high-frequency measurements using covariances in consecutive 20 min time intervals. The (virtual) sensible heat flux *H*_*S*_, derived from the covariance of (virtual) temperature and vertical wind speed, quantifies the turbulent heat transfer between the surface and the atmosphere. The momentum flux *τ*, derived from the covariance of horizontal and vertical wind speed, characterizes the transfer of momentum due to wind shear. These turbulent fluxes are computed as:

6 $${H}_{S}=\overline{\rho }\cdot {{\rm{c}}}_{{\rm{p}}}\cdot \overline{w{\prime} {T}_{v{\prime} }}$$

7 $$\tau =-\overline{\rho }\cdot \overline{w{\prime} U{\prime} }$$

The overline denotes an average of the subrecord, while the prime signifies the turbulent fluctuation $\left(U{\prime} =U-\overline{U}\right)$. The mean air density $\overline{\rho }$ is calculated based on the 2 m inflow measurements, and c_p_ = 1006 J kg ^−1^ K ^−1^ is the specific heat capacity of air. The covariance $\overline{w{\prime} U{\prime} }$ includes fluctuations from all horizontal wind components as suggested by Weber^18^ for the friction velocity. Covariances are computed for parameters at the 7 m height level from detrended, non-overlapping 20 min time segments. The 7 m height level was selected since this measurement height was least impacted by the solar collector field (the maximum collector height is ∼5.5 m), thereby providing the most representative measurements of undisturbed atmospheric wind conditions. Ogive analyses^19,20^ have demonstrated that a 20 min window effectively captures the major frequency contributions to the covariances and confirm their convergence within this time frame for the majority of cases. Covariances also allow for the calculation of the Obukhov length and the flux Richardson number, which are alternative measures of dynamic stability based on high-frequency measurements at only one height. The Obukhov length *L* is defined by the vertical turbulent fluxes of heat and momentum

8 $$L=-\frac{{u}_{\ast }^{3}\cdot {\overline{T}}_{{\rm{ref}}}}{\kappa \cdot g\cdot \overline{{w}^{{\prime} }{T}_{v}^{{\prime} }}}$$

where ${u}_{\ast }=\sqrt{| \overline{{U}^{{\prime} }{w}^{{\prime} }}| }$ is the friction velocity as calculated for the momentum flux and *κ* = 0.4 is the von Kármán constant. The 2 m temperature serves as *T*_ref_, since this variable is available for the entire measurement period. The sign of *L* indicates the stability of the atmosphere: positive values correspond to stable conditions and negative values to unstable conditions. The flux Richardson number *R*_*f*_ is determined by

9 $${R}_{f}=\frac{g}{{\overline{T}}_{{\rm{ref}}}}\cdot \frac{\overline{{w}^{{\prime} }{T}_{v}^{{\prime} }}}{\overline{{u}_{E}^{{\prime} }{w}^{{\prime} }}\cdot (\Delta {\overline{u}}_{E}/\Delta z)+\overline{{v}_{N}^{{\prime} }{w}^{{\prime} }}\cdot (\Delta {\overline{v}}_{N}/\Delta z)}$$

with the covariance of the eastward wind component *u*_*E*_ and vertical wind $\overline{{u}_{E}^{{\prime} }{w}^{{\prime} }}$ and the wind gradient $\Delta \overline{{u}_{E}}/\Delta z$ between 3.5 m and 7 m (analog for *v*_*N*_).

For all sonic anemometers on the wake and inflow masts, turbulent integral length scales are also calculated. Integral length scales ${\mathscr{L}}$ describe the characteristic size of energy-containing eddies in a turbulent flow. Here, they are calculated from the autocorrelation function *A* of a continuous time series from a point measurement (as provided by the sonic wind speeds). *A*_*w*_ for the vertical wind component *w*(*t*) results from

10 $${A}_{w}({t}^{\ast })=\frac{\overline{w{(t)}^{{\prime} }\cdot w{(t+{t}^{\ast })}^{{\prime} }}}{{\sigma }_{w}^{2}}$$

where *A*(t^*^) represents the autocorrelation function at time lag t^*^. The integral of the autocorrelation curve first provides the integral time scale ${{\mathscr{T}}}_{w}$, which can be transferred into a length scale by ${{\mathscr{L}}}_{w}=\overline{U}\cdot {{\mathscr{T}}}_{w}$ using Taylor’s hypothesis of “frozen turbulence” transported by the mean wind^21^. The detrended 20 min time series of *U* and *w* are shifted by time lags t^*^ between 0 s and 20 min, and for each lag, the autocorrelation coefficient *A* of the original with the shifted time series is calculated. As an approximation for the integral under the *A*_*w*_(t^*^) curve, the curve is assumed to be an exponential function, and the intercept $A{({t}^{\ast })}_{w}=1/e\approx 0.368$ at ${t}^{\ast }={{\mathscr{T}}}_{w}$ yields the integral time scale^19,21^. If the time scale is larger than the time series segment, the result is excluded. We find that the exponential fit method yields the most robust results, although estimating length scales from time series is challenging and many large length scales are missing because of the limited length of the time series segments. The same approach as discussed for *w* is applied to derive the turbulent length scales ${{\mathscr{L}}}_{U}$ for the horizontal wind speed and ${{\mathscr{L}}}_{uE}$ and ${{\mathscr{L}}}_{vN}$ for the eastward and northward component, respectively.

The postprocessed measured and derived data are stored at 20 Hz resolution with the covariance-derived values added at 20 min intervals. Variables in the processed dataset are listed in Table 2. Additionally, 1 min averaged data are provided with peak values and standard deviations of wind speeds in these intervals.

**Table 2:** Variables included in the published inflow and wake mast datasets.

Variable	Symbol	Description	Unit
**Inflow mast**			
u_*X*m	*u*_*E*_	Eastward wind component at *X* m height	m/s
v_*X*m	*v*_*N*_	Northward wind component at *X* m height	m/s
w_*X*m	*w*	Upward wind component at *X* m height	m/s
Ts_*X*m	*T*_*s*_	Sonic temperature at *X* m height (not calibrated)	°C
wdir_*X*m	WD	Wind direction at *X* m height	°
wspd_*X*m	*U*	Horizontal wind speed at *X* m height	m/s
p	*p*	Pressure at 2 m height	hPa
Temp_*X*m	*T*	Temperature at *X* m height	°C
RH	RH	Relative humidity at 2 m height	%
TKE_*X*m	TKE	Turbulence kinetic energy at *X* m height	m^2^/s^2^
TI_U_*X*m	TI_*U*_	Turbulence intensity of horizontal wind at *X* m height	—
TI_w_*X*m	TI_*w*_	Turbulence intensity of vertical wind at *X* m height	—
TI_uE_*X*m	TI_*uE*_	Turbulence intensity of eastward wind at *X* m height	—
TI_vN_*X*m	TI_*vN*_	Turbulence intensity of northward wind at *X* m height	—
ls_U_*X*m	${{\mathscr{L}}}_{U}$	Horizontal length scale at *X* m height	m
ls_w_*X*m	${{\mathscr{L}}}_{w}$	Vertical length scale at *X* m height	m
ls_uE_*X*m	${{\mathscr{L}}}_{uE}$	Length scale in east direction at *X* m height	m
ls_vN_*X*m	${{\mathscr{L}}}_{vN}$	Length scale in north direction at *X* m height	m
Ri_b	Ri_*b*_	Bulk Richardson number	—
Ri_f	R_*f*_	Flux Richardson number	—
H_S	*H*_*S*_	Virtual sensible heat flux	W/m^2^
Tau	*τ*	Momentum flux	kg m/s
L	*L*	Obukhov length	m
**Wake mast** ***Y*** **(*Y* = 1, 2, or 3)**			
m*Y*_u_*X*m	*u*_*E*_	Eastward wind component at *X* m height	m/s
m*Y*_v_*X*m	*v*_*N*_	Northward wind component at *X* m height	m/s
m*Y*_w_*X*m	$w$	Upward wind component at *X* m height	m/s
m*Y*_Ts_*X*m	*T*_*S*_	Sonic temperature at *X* m height (not calibrated)	°C
m*Y*_wdir_*X*m	WD	Wind direction at *X* m height	°
m*Y*_wspd_*X*m	*U*	Horizontal wind speed at *X* m height	m/s
m*Y*_TKE_*X*m	TKE	Turbulence kinetic energy at *X* m height	m^2^/s^2^
m*Y*_TI_U_*X*m	TI_*U*_	Turbulence intensity of horizontal wind at *X* m height	—
m*Y*_TI_w_*X*m	TI_*w*_	Turbulence intensity of vertical wind at *X* m height	—
m*Y*_TI_uE_*X*m	TI_*uE*_	Turbulence intensity of eastward wind at *X* m height	—
m*Y*_TI_vN_*X*m	TI_*vN*_	Turbulence intensity of northward wind at *X* m height	—
m*Y*_ls_U_*X*m	${{\mathscr{L}}}_{U}$	Horizontal length scale at *X* m height	m
m*Y*_ls_w_*X*m	${{\mathscr{L}}}_{w}$	Vertical length scale at *X* m height	m
m*Y*_ls_uE_*X*m	${{\mathscr{L}}}_{uE}$	Length scale in east direction at *X* m height	m
m*Y*_ls_vN_*X*m	${{\mathscr{L}}}_{vN}$	Length scale in north direction at *X* m height	m

### Lidar measurements of winds

To characterize flow conditions directly above the solar collector field, a Galion G4000 pulsed Doppler wind lidar^22^ was installed approximately 70 m south of the inflow meteorological (met) tower. This scanning lidar was installed on top of two shipping containers at approximately 6 m above the ground (Fig. 1) with a leveled top surface. We used a Ushikata Transit to align the Lidar in the field along the cardinal compass directions.

The Galion lidar works by emitting laser pulses into the atmosphere and analyzing the backscattered light to measure the Doppler shift, providing information about line-of-sight (LOS) wind speed in the direction of the laser beam. Along the beam, the lidar provides a spatial resolution corresponding to the non-overlapping, 18 m range gate lengths. Usable data can be obtained between ≈ 70 m distance from the lidar (the “blind zone”) to the configured maximum scanning distance of 612 m. The lidar at NSO was configured to do two types of scans in the horizontal plane above the collectors: a 360 ° horizontal scan (or Plan Position Indicator–PPI scan, used between June 2022 and September 2022) and a 0.4 Hz stare scan (or fixed LOS scan, between April 2023 and May 2023). The purpose of the 360 ° horizontal scan was to investigate the impact of parabolic troughs on wind conditions within the plant at a height above the troughs. This pattern performed full horizontal 360 ° scans in 1 ° increments every 10 minutes and yielded reliable data within an opening angle of ± 30 ° about the mean wind direction. During a stare scan, the lidar operates and scans at a fixed azimuth and elevation angle, and the fixed laser beam captures temporal variations at a higher temporal resolution. The stare scan in the west-east direction over the solar collector field aimed to characterize turbulence conditions along the west-east scanning direction during western wind events. Using the ≈ 0.4 Hz LOS wind speed within each range gate, the variance in wind speed can be determined. Note that resulting variances might be underestimated due to the limited probe volume averaging. Further, western wind directions are rare at NSO, limiting available data for the stare scan period, despite scientific interest. The technical aspects of the two different scan patterns are listed in Table 3.

**Table 3:** Technical details of the two types of lidar scan patterns.

Range Gate Length	Numbers of Range Gates	Frequency	Scanning Angle (Azimuth)	Measurement Height	Measurement Period
**1) 360° Horizontal Scan**					
18 m	34	∼10 min per scan	0–360°	∼6 m	June 2022–Sept 2022
**2) Stare Scan (west-east direction)**					
18 m	34	∼0.4 Hz	90°	∼6 m	April 2023–May 2023

The dataset (variables are listed in Table 4) includes the LOS wind speed along the laser beam (“Doppler”) and information about the direction of the beam, characterized by azimuth (Az, the angle in the horizontal plane starting at north) and elevation (El, the angle above the horizontal plane). For the horizontal scan, Az varied between 0° and 360° and El was constant at 0°, whereas both Az and El were constant at 90° and 0°, respectively, for the stare scan. Before analyzing wind speed data, low-quality data need to be filtered by calculating the signal-to-noise ratio (SNR) and eliminating data samples with low SNR. The SNR is calculated from the power intensity PI (provided in the lidar data) using:

11 $${\rm{SNR}}=10\cdot {log}_{10}({\rm{PI}}-1)$$

**Table 4:** Variables included in the published lidar datasets.

Variable	Symbol	Description	Unit
**Lidar**			
Ray_time		Measurement time (UTC, yyyy-mm-dd hh:mm:ss)	—
Range_gate		Range gate number	—
Doppler		Line-of-sight wind speed	m/s
Doppler_filtered		Line-of-sight wind speed, filtered for bad data quality	m/s
Intensity	PI	Power intensity	—
Azimuth	Az	Azimuth angle of the lidar beam in the horizontal plane starting at north	°
Elevation	El	Elevation angle of the lidar beam above the horizontal plane	°
Pitch		Instrument alignment around the side-to-side axis	°
Roll		Instrument alignment around the front-to-back axis	°

For our data, we recommend using an empirical threshold of SNR = −19 such that lidar data with SNR > −19 is kept for further analysis while the rest is discarded. However, the threshold might have to be adjusted for specific cases. The filtered LOS wind speed is included in the dataset (“Doppler_filtered”). Note that pitch and roll represent the lidar instrument alignment. These values were not changed during the course of the measurement campaign, and no alignment correction was made in the dataset. Lastly, the lidar dataset contains some short interruptions leading to multi-hour gaps in data during the summer months due to the lidar overheating.

### Measurements of structural loads

#### General setup

**Fig. 4:**
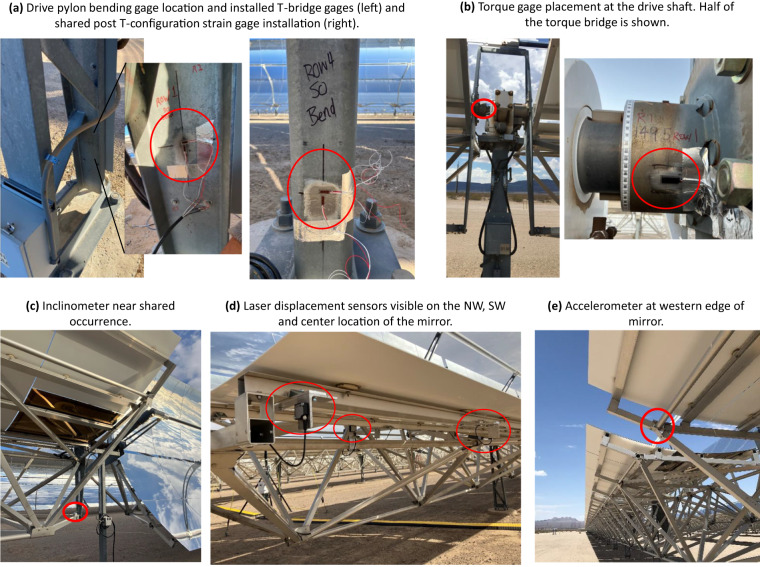
Pictures of the load sensor installations.

In addition to the meteorological mast measurements, structural loads at the parabolic trough collectors were measured between November 2022 and June 2023. The sensors were installed in rows 1, 2 and 4 on the half-SCA close to the met masts. The individual load sensors and their processing routines are explained in more detail in the following sections. The sensor locations on the space frame or trough mirrors are depicted in Fig. 4. The individual sensor models are listed in Table 5. In addition to loads sensors, a GPS time stamp was sampled as well as the cup anemometer wind speed signal at 15 m height. The data from all load sensors were collected in one central data acquisition system (DAS). The DAS is based on highly configurable National Instruments (NI) hardware and software. A local host computer in the plant control room and a NI PXI system (composed of chassis, controller, and modules) were used to control and interface with three chassis that were distributed across trough rows 1, 2, and 4. Communication between the PXI system and the chassis was established through fiber optics, employing EtherCAT media converters. This setup allowed for reliable and efficient data transfer between the components.

**Table 5:** Instrumentation for the structural loads measurements.

Measured quantity	Instrument	Model and resolution
Torque moment at Drives	Strain gages	Vishay LEA-06-W125F-350/3 R, ± 0.4%Ω resolution
Support Structure Bending Moments	Strain gages	Vishay LWK-06-W250B-350, ± 0.4%Ω resolution
Dynamic Tilt	Inclinometer	2GiG BH-1800-000-2M, 0.05° accuracy
Structural Dynamics	Accelerometers	Silicon Designs 2460-10, 3.0e-6 g rms resolution
Mirror Deflections	Laser displacement sensor	Baumer OM30-L0350, 120 *μ*m resolution

As a first signal processing step, calibration slopes and offsets were applied to the raw data in the DAS so that the resulting files contained data in engineering units, with the exception of the strain gage signals where the conversion factors were applied in postprocessing. The collected data were saved to the DAS PC. Data were regularly uploaded from the PC to a file transfer protocol site hosted by NREL and then routinely downloaded and saved to the NREL server for postprocessing. During the test campaign, there were several periods where loads data were lost, probably due to interrupted communication from the PXI to the EtherCAT data chain. These events required the DAS to be manually reinitialized to resume communications and data collection. The data acquisition process involved a data scan rate at 1 kHz, while data were sampled and stored at a reduced rate of 20 Hz, applying an anti-aliasing filter in the NI signal conditioning.

For postprocessing, the loads data had to be time-synchronized with the measured wind data. This was done with the GPS time stamp recorded in both data streams. For loads measurements before February 23, 2023, the loads GPS sensor did not receive satellite signals. Therefore, the time stamp of the data logger had to be corrected manually by correlating the wind speed signal at 15 m height, which was recorded in both the inflow mast and loads datasets. This manual time stamp correction provides an accuracy of below 1 s for the affected period. As a first general data processing step, outliers and nonphysical values were excluded. Variables in the processed dataset are listed in Table 6. All published loads data are provided as time series at 20 Hz resolution and additionally at an averaged 1 min resolution. These sampling frequencies match the met mast frequencies and enable straightforward merging of the datasets. Additional parameters in the 1 min loads dataset are maxima, minima, and standard deviations for all loads variables for users interested in peak values or dynamics. The original 20 Hz time series can also be used to calculate loads spectra.

**Table 6:** Variables included in the published loads datasets.

Variable	Symbol	Description	Unit
**Loads at row** ***Z*** **and location** ***loc*** **(*Z* = 1, 2, or 4;** ***loc* = DO, SO, or Mid)**			
R*Z*_*loc*_Bending	*M*_*B*_	Bending moment at DO or SO	kN m
R*Z*_DO_Torque	*M*_*y*_	Torque moment at DO	kN m
R*Z*_*loc*_Accel_X	*a*_*x*_	Acceleration at space frame on western edge, perpendicular to mirror plane	g
R*Z*_*loc*_Accel_Y	*a*_*y*_	Acceleration at space frame on western edge, in mirror plane	g
R*Z*_Disp_*pos*	d	Mirror displacement at westernmost, mid panel (*pos* = NW, NE, SW, SE or Center), zero-value subtracted	mm
R*Z*_Disp_*pos*_raw	*d*_0_	Mirror displacement as above, absolute value	mm
R*Z*_*loc*_Tilt	*θ*	Calibrated tilt of space frame at DO, Mid, or SO	°
R*Z*_*loc*_Tilt_raw	*θ*_0_	Uncalibrated tilt of space frame at DO, Mid, or SO	°
projected_sun_angle	*β*_*EW*_	Projected sun angle in the East-West plane	°
Anemometer	*U*	Wind speed at 15 m height (same as in wind data)	m/s
R*Z*_*loc*_C_Bending	*C*_*mb*_	Bending moment coefficient at DO or SO	—
R*Z*_DO_C_Torque	*C*_*my*_	Torque moment coefficient at DO	—
R*Z*_*loc*_Cfx	*C*_*fx*_	Drag force coefficient at DO or SO	—

#### Individual sensors

##### Support structure bending moments

Bending moments of the trough support structure were measured at rows 1, 2, and 4 at both the SO and DO pylons. The bending moments were measured using strain gages in a full bridge T-configuration that is shown in Fig. 4a. The strain gages measure deformations that are calibrated to provide an experienced moment. The DO and SO pylons are built differently (Fig. 2): the DO pylon has a triangular truss construction and the SO pylon is a simple bending beam; therefore, the installation of the gages differed at the DO and SO pylons. For both, the gages were placed near the lower third of the exposed pylon and at least one width of the tube/web away from the concrete foundation. For the drive pylon, the gages were placed at *H*_gage_ = 0.480 m from the foundation and on the inside of the c-channel. This location was selected to avoid interference from electrical enclosures attached to the outside of the pylon. The shared occurrence gages were placed at *H*_gage_ = 0.175 m from the foundation and on the inside of the square tube. At this location, the gages were approximately one cross section away from the base plate.

**Fig. 5:**
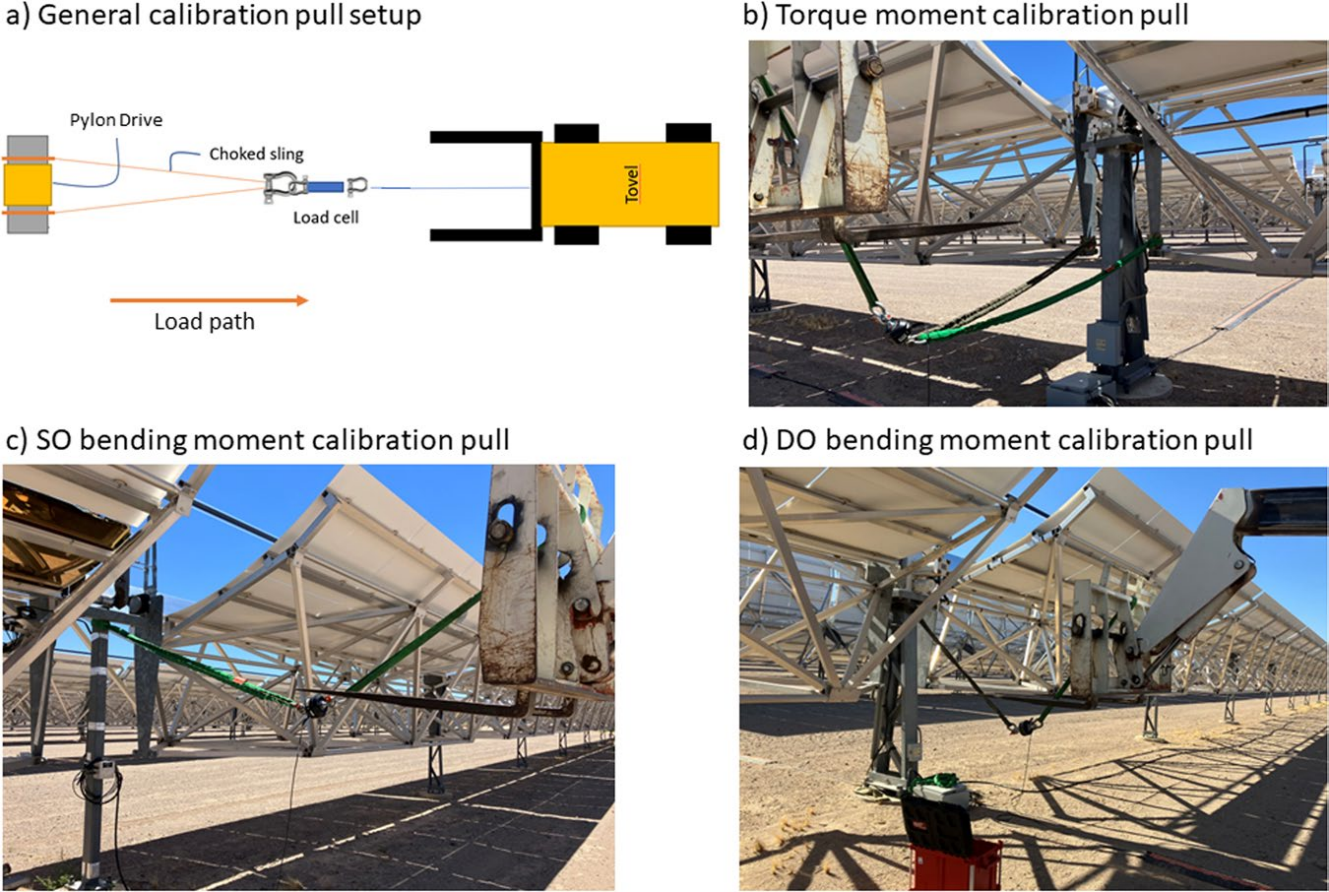
Schematic and pictures of strain gage calibration pulls.

To arrive at engineering units of the strain signals, the strain signal offset was subtracted from the raw strain data. The strain offsets were determined using the average strain values over the entire dataset, filtered for low wind speeds and bin-averaged over tilt angles less than ±70° to the upward position. Next, the signal was multiplied by the scale factor. The scale factors were determined through a calibration pull of the instrumented structural component, using a telehandler with slings, shackles, and a reference load cell in line with the rigging (Fig. 5). Load was incrementally applied by slowly retracting the boom. This calibration provided moment values for each SO and DO pylon.

To convert the bending moments at the measurement location to overturning bending moments *M*_*B*_ (the actual bending moment about the foundation axis parallel to the *y* direction), the following correction was applied with *H*_c_ = 0.279 m (Fig. 2):

12 $${M}_{B}={M}_{B,meas}\cdot \frac{{H}_{c}}{{H}_{c}-{H}_{{\rm{gage}}}}$$

The bending moments can also provide a drag force in the *x*-direction:

13 $${F}_{x}={M}_{B}/{H}_{c}$$

The measured moments are defined right-handed positive along the *y*-axis (Fig. 2). Because of the different layouts of the pylons, bending moments behaved differently at SO and DO. At the simple-bending-beam SO pylon, the measured moments can be assumed to be actual bending moments. In contrast, at the triangle-truss-structure DO pylon, the measured moments show some overlaying torque moment characteristics (resulting from tension and compression in the truss structure) in addition to pure bending characteristics. As a result, the bending moments and drag forces at the DO should be treated with caution, but are still included in the dataset for completeness.

To compare the forces and moments to other studies, the loads were transformed to load coefficients *C*_*mb*_ for the bending moments and *C*_*fx*_ for the drag forces:

14 $${C}_{mb}=\frac{{M}_{B}}{\frac{\overline{\rho }}{2}{\overline{U}}^{2}\cdot {L}_{{\rm{segment}}}\cdot W\cdot {H}_{c}}$$

15 $${C}_{fx}=\frac{{F}_{x}}{\frac{\overline{\rho }}{2}{\overline{U}}^{2}\cdot {L}_{{\rm{segment}}}\cdot W}$$

where the 60 s mean air density $\overline{\rho }$ and mean horizontal wind speed $\overline{U}$ were determined from the met mast dataset. *L*_segment_ = 8 m is the spanwise length of a mirror segment, and $W=5$ m is the aperture width. The moment coefficient *C*_*mb*_ represents the bending moment about the foundation of the pylons, whereas the horizontal drag force coefficient *C*_*fx*_ is calculated from the measured bending moment by dividing by the lever length (hinge height). The load coefficient definitions are adapted from Hosoya *et al*.^9^. Coefficients were calculated for data with wind speeds (inflow mast at 3.5 m height) greater than 3 m/s to avoid excessive values resulting from low values in the denominator. For 1 min averaged data, the coefficients were calculated with the 1 min means of moments, wind speed, and air density. The load coefficients for wind speeds from eastern directions should be treated with caution because the wind field is impacted by the entire trough field before reaching the instrumented rows, and the coefficients might not be reasonable. For the sake of completeness, they are still included in the published data.

##### Drive torque moments

Torque moments at the drive location were measured on rows 1, 2, and 4 using strain gages configured as a full torque bridge. The strain gages were placed on the driveshaft south of the drive actuator (Fig. 4b). There was limited free length of the drive shaft to allow for strain gage installation. The gages were instead installed on the shaft of the hub that attaches to the drive shaft. In this case the installation did not meet the recommended distance of one cross-section diameter of the drive shaft away from any possible stress riser or discontinuities. The gages were oriented to 90° away from the shaft keyway to avoid discontinuities. Torque moments were calibrated in the field analog to the bending moments using the mean of low-wind, tilt-bin-averaged offsets and a calibration pull for slope calibration.

The torque moments are defined right-hand positive about the pivot axis *y* of the parabolic troughs (Fig. 2). The torque moment distribution over the torque axle has its maximum at the drives (corresponding to the measured moment) and decreases linearly to zero near the SO end of the torque axle. Similar to the bending moment, the torque moments have been transformed into moment coefficients *C*_*my*_:

16 $${C}_{my}=\frac{{M}_{y}}{\frac{\overline{\rho }}{2}{\overline{U}}^{2}\cdot {L}_{{\rm{panel}}}\cdot {W}^{2}}$$

where *L*_panel_ = 49 m is the spanwise length of an entire mirror panel (one-half of an SCA).

##### Dynamic tilt

Dynamic tilt angles were measured at row 1, row 2, and row 4. Each instrumented trough row included three dynamic inclinometers located near the bottom of the space frame with one unit at the SO (Fig. 4c), one unit at the DO, and one centrally located (“Mid”) on the trough section. The inclinometers were installed so that when the trough is flat, the response is a zero-degree reading. Positive degrees are measured when the trough is facing east (in the morning) and negative degrees when facing west (in the evening). The stow position is 120 ° (30 ° below the eastern horizon), but occasionally the troughs are brought into a stow position of 150 ° or 90 °.

The trough’s “nominal” tilt angle can be calculated from the sun angle according to the NREL Sun Position Algorithm (https://midcdmz.nrel.gov/solpos/spa.html), which is available through a python API^23^. The nominal tilt angle *θ*_nom_ is calculated through the algorithm described in Anderson & Mikofski^24^, which projects the sun position into the transversal (east-west) plane using the elevation and azimuth angles. The dataset includes this transversal sun angle *β*_EW_ at the NSO location.

Measured tilt angles *θ*_0_ at horizontal trough positions for each sensor were calibrated in the field using digital protractors. However, the inclinometers measure the angle of the space frame at the measurement location instead of the actual angle of the parabolic troughs. Comparing measured tilt angles against expected trough angles (from the sun angle), we found consistent offsets. We provide a corrected tilt angle that accounts for these offsets. For this, we define the tilt angle error *ε* along the transversal (east-west) plane as *ε* = *θ*_0_ − *θ*_nom_. Using a known angular position—stow at 120°—we calculate an average calibration constant for each sensor in the 1 min data and subtract this constant from the data. To find this calibration constant, we extract data that satisfy the following conditions:

- Measured tilt angle equals 120° ± 1°.
- Wind speeds are less than 1 m/s.
- Maximum tilt angle error ($| \varepsilon | =| {\theta }_{0}-12{0}^{\circ }|$) is less than 1.6° because tilt angle errors greater than this value indicate operational settings outside of stow.

We apply this filtering to the data and calculate average tilt angle errors for the time periods before and after December 22, 2022, separately because the NSO operators performed a calibration on this date and recalibrated the drive motors of the parabolic troughs.

The filtered dataset consists of 5,159 data points before December 22, 2022, and 3,747 data points after December 22, 2022. The average tilt angle errors are calculated for each sensor and are shown in Table 7. We subtract the average tilt angle error for each sensor from the tilt data before and after December 22, 2022, to calculate the calibrated tilt angle $\theta ={\theta }_{0}-\overline{\varepsilon ({\theta }_{{\rm{nom}}}=12{0}^{\circ })}$, which reduces the average tilt angle error. For example, in row 1 at the DO, the average tilt angle error of all daytime data after December 22, 2022, and below the error threshold of 1.6° is reduced from −1.51° to 0° after the calibration. The dataset contains both “raw” tilt angles as measured by the inclinometers *θ*_0_ as well as calibrated values *θ* with the average stow position error subtracted (Table 6).

**Table 7:** Average tilt angle error $\bar{\varepsilon ({\theta }_{{\rm{nom}}}=12{0}^{\circ })}$ from the filtered data at stow position during low wind speeds used as the calibration constants.

	Row 1			Row 2			Row 4		
	DO	Mid	SO	DO	Mid	SO	DO	Mid	SO
$\overline{\varepsilon }$ (°), pre-Dec-22-22	−1.502	−0.804	−0.956	0.504	0.036	0.850	0.603	0.141	0.810
$\overline{\varepsilon }$ (°), post-Dec-22-22	−1.518	−0.842	−0.981	0.443	0.014	0.811	0.598	0.126	0.822

##### Mirror deflections

**Fig. 6:**
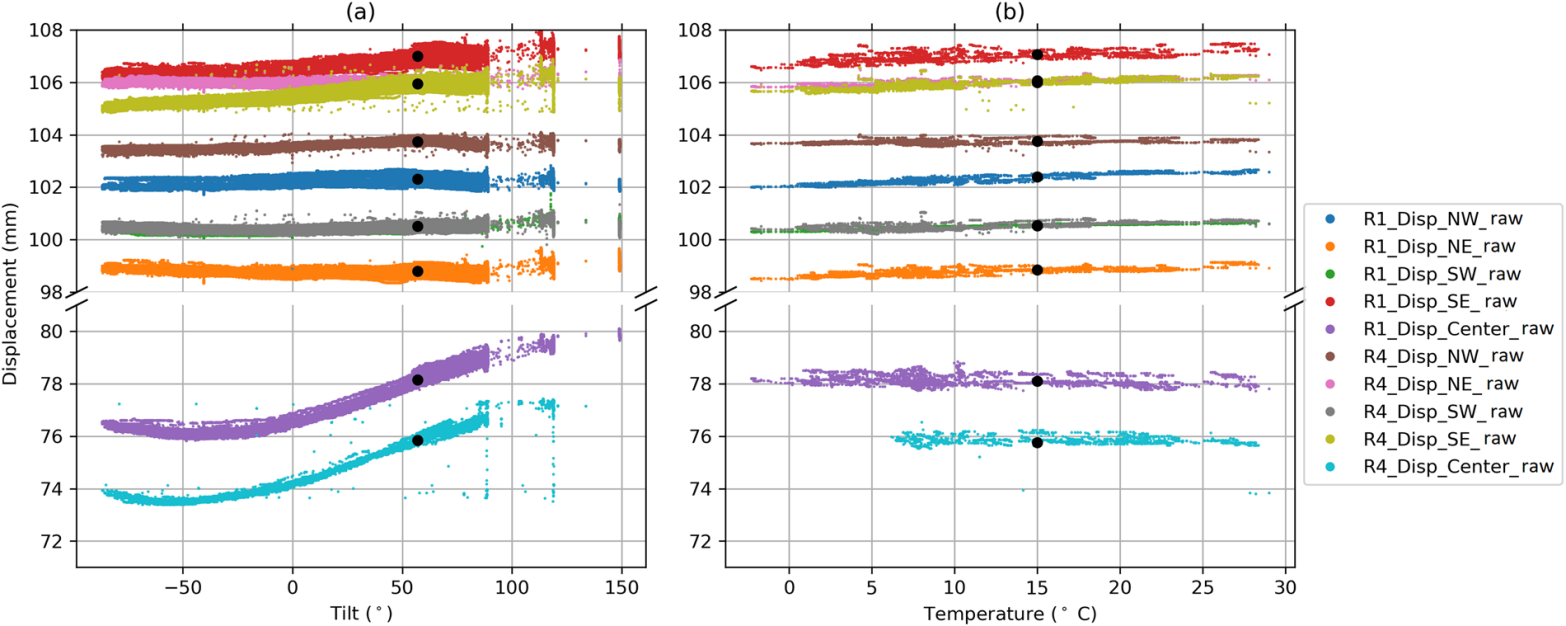
Influence of gravity (represented by the tilt angle of the troughs) and temperature on raw mirror displacements measured by the individual sensors before calibration. Each data point represents a 1 min average during the entire measurement period. The calibration points (vertical mirror position and 15 °C) are shown as black dots.

Mirror deflections were measured using laser displacement sensors on two single mirrors on the western edge of trough rows 1 and 4. Each of the two instrumented mirrors is located at the center span of the trough section south of the intermediate pylon. Five laser displacement sensors at each mirror were placed on the space frame behind the mirror to measure the distance between the rear surface of a mirror near each of its corners (NW, SW, NE, SE; in proximity to the connection points) and at the mirror center (Fig. 4d). The sensors attached to the space frame provided the relative distance between the mirrors and the space frame. As a result, the measured displacements represented a combination of the support structure deformation and the deformation of the glass mirror itself. This relative distance was between 75 mm and 80 mm for the central sensors and between 98 mm and 108 mm for the sensors at mirror corners (Fig. 6). To find a “zero-position” for the displacement measurements, we filter cases with low winds (<3 m/s) and vertically oriented mirrors (to exclude gravity influences as shown in Fig. 6a). This vertical mirror position occured in the late mornings before solar noon. Unfortunately, the opposite position (180° rotated) was never taken in normal operation. We noticed that the described zero-position is not a constant displacement value but depends on the ambient temperature. The relation of relative displacement to temperature is nearly linear (Fig. 6b) with a positive slope for the corner sensors and a slightly negative slope for the center sensors, but with a spread below 1 mm for all sensors. We define a zero-displacement at the vertical mirror position and at an ambient temperature of 15 °C to study wind, temperature and gravity influences on mirror deformations. The dataset contains both “raw” relative distances between mirror and space frame as well as adjusted values with the zero-displacement subtracted (Table 6).

##### Accelerations at the space frame

Accelerometers were installed on rows 1, 2, and 4 on the northern and southern end of one-half SCA near the DO and SO pylons. They were attached to the space frame on the western edge of the trough (Fig. 4e). The accelerometer sensors were attached to the spaceframe behind the mirrors and rotate with the assembly. The biaxial measurement directions of each accelerometer are orthogonal (with the local *y*-axis in the plane of the mirror and with the local *x*-axis perpendicular to the mirrors). The frequency range allowed for a wide range of dynamics measurement, down to the static acceleration, or gravity. The static acceleration due to gravity changes from 1 g to 0 g as the sensor moves from a vertical orientation (aligned with gravity) to a horizontal orientation, and therefore also provides a measure of the trough angle as this is proportional to the tilt. The acceleration signals for low-wind cases were corrected to be within +1 g and −1 g, when the trough was rotating. These corrections were subsequently applied to all data irrespective of the wind speeds.

## Data Records

All datasets are available at the OEDI (Open Energy Data Initiative) data repository at 10.25984/2001061^25^. The datasets are divided into seven categories: inflow_mast_1min, inflow_mast_20Hz, wake_masts_1min (the three combined wake masts), wake_masts_20Hz, loads_1min, loads_20Hz, and lidar. The data files are in a folder structure containing the category, year, month, and day. All data records are saved in the Parquet file format. The Parquet data format has been selected for various reasons: Compared to other file formats such as CSV or NetCDF, the Parquet format reduces storage space requirements and facilitates fast and efficient read and write operations, making it well-suited for handling large datasets. Since all of our data are 1-dimensional time series data, the complexity of the NetCDF format was deemed unnecessary. Parquet files offer universal compatibility with various programming languages such as Python, Matlab, and R. Furthermore, the column structure of the Parquet format allows to selectively read only the desired columns. Example Python scripts to read the datasets in Parquet file format are provided at the OEDI repository^25^. The data structure consists of daily Parquet files, where separate files are provided for the 20 Hz and 1 min resolutions (for loads and met masts). The naming convention for these files follows the pattern: *Type*_*resolution*_*YYYY*-*MM*-*DD*_00h_to_*YYYY*-*MM*-*DD*_00h.parquet, where *YYYY*-*MM*-*DD* represents the date of the daily file that contains data from 00:00 UTC to 00:00 UTC the next day (times may vary when there are data gaps). The *Type* can be one of four options: *Inflow_Mast*, *Wake_Masts*, *Loads*, or *Lidar*, while the *resolution* can be either *1 min* or *20 Hz* (the lidar files contain no resolution). All time stamps in the data are in UTC format. For example, the file name for 1 min loads data on November 19, 2022, would be *Loads*_*1min*_*2022-11-19*_*0h*_*to*_*2022-11-20*_*0h.parquet*. For the lidar data, each file includes one complete lidar scan, and the file names include the hour, minute, and second of start and end time. An example is *Lidar_2023-04-02_19-00-17_to_2023-04-02_19-00-17.parquet*. For the lidar stare scan, only the data for 15 selected western wind periods are archived, whereas continuous lidar data are available for the horizontal scan period (June 2022–September 2022). Table 2, Table 4, and Table 6 list all variables in the met mast, loads, and lidar datasets with their respective formula symbol, a description, and units. When a single instrument on a met mast was not available on a specific day, the respective data column is filled with NaNs. In addition to these listed variables, the 1 min met mast datasets include maximum and standard deviation values of wind speed, derived in 1 min windows. Furthermore, the loads datasets contain maximum, minimum, and standard deviation values of 1 min windows for all loads variables.

## Technical Validation

### Mast measurements of winds

**Fig. 7:**
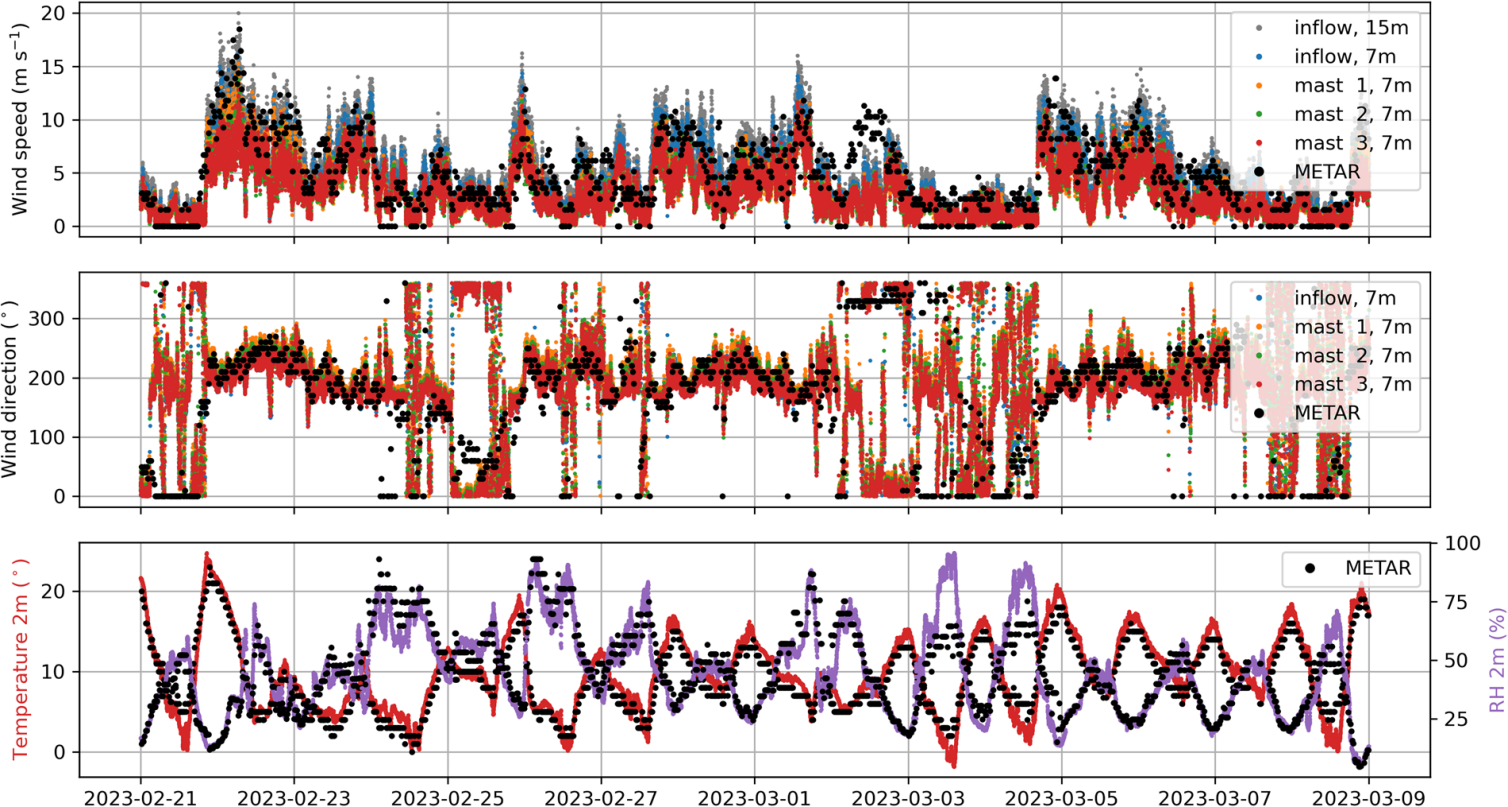
Verification of the met mast measurements at Nevada Solar One with METAR data from the Boulder City Municipal Airport.

The met mast measurements at NSO are compared to the METAR (METeorological Aerodrome Report) observations from Boulder City Municipal Airport, located approximately 20 km northeast of NSO. These data are provided by the Iowa Environmental Mesonet of Iowa State University (https://mesonet.agron.iastate.edu/request/download.phtml). Note that differences in observations can arise due to spatial, orographic, and temporal variations. However, this comparison serves to verify the accuracy of the coordinate transformations applied to the sonic anemometers. Figure 7 presents a comparison of wind speed and direction measurements across all met mast heights. Additionally, the temperature and RH measurements from the inflow mast are included in the comparison. The results indicate a good qualitative agreement between the met mast measurements at NSO and the METAR observations. This agreement lends confidence to the correctness of the coordinate transformations used for the sonic anemometers and validates the reliability of the measured wind speed, wind direction, temperature, and RH data.

### Lidar measurements of winds

**Fig. 8:**
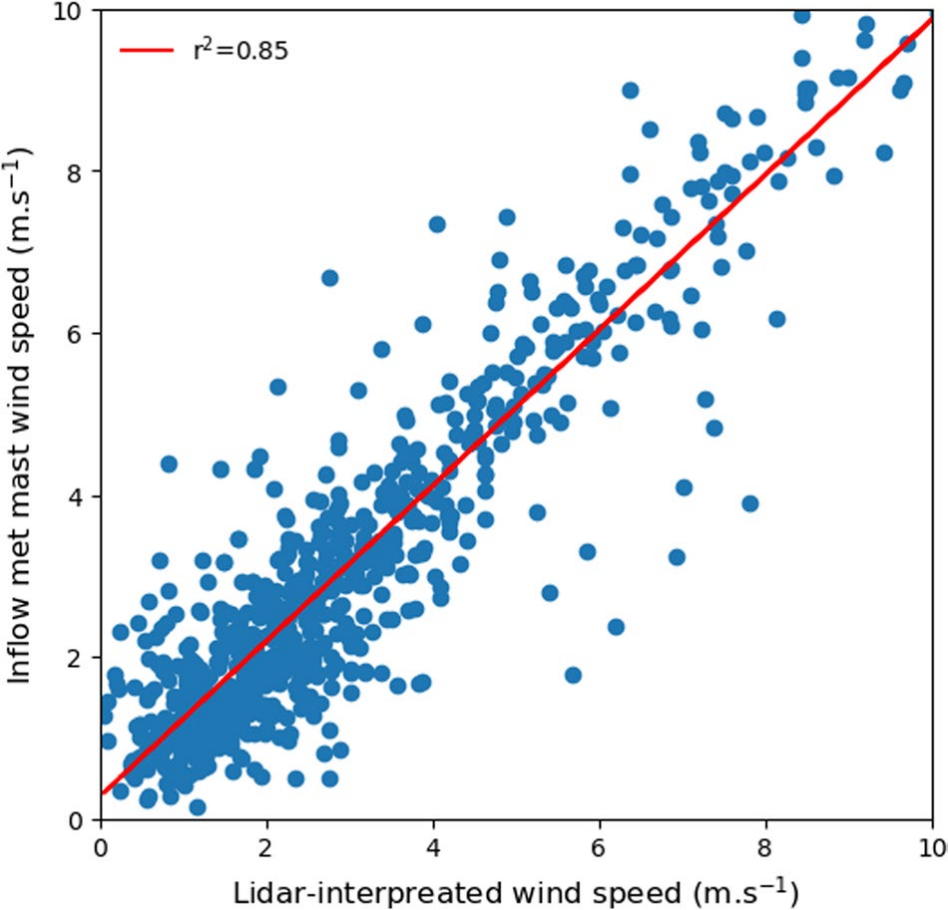
Wind speed comparison between the lidar and the inflow met mast at 7 m height.

The wind speeds determined by the lidar are verified with the inflow met tower measurements. Since the lidar measures wind speed at about 6 m above the ground, the measurement at 7 m height from the inflow met tower is used for qualitative validation. The LOS wind speed from the lidar is first converted into true wind speed using the wind direction from the inflow met mast before conducting the comparison. Overall, the lidar-interpreted wind speed matches qualitatively well with the measurements from the inflow met mast (coefficient of determination r^2^ = 0.85; Fig. 8), suggesting good agreement between these two independent instruments. Note that r^2^ in this case is not greater than 0.9, which is a common threshold lidar-mast comparisons. This is most likely because the distance between lidar and inflow met tower is too close to the measurement blind zone of the lidar (four range gate numbers, or approximately 72 m). More lidar-associated analysis will be presented in a follow-on study.

## Usage Notes

An example Jupyter notebook script written in python is provided at the OEDI repository^25^ to read the presented datasets.

## Data Availability

The Python processing routines for the met masts, lidar and loads data are publicly available at https://github.com/NREL/NSO_processing_scripts.
